# GR-mediated transcriptional regulation of m^6^A metabolic genes contributes to diet-induced fatty liver in hens

**DOI:** 10.1186/s40104-021-00642-7

**Published:** 2021-12-07

**Authors:** Yue Feng, Yanlin Li, Wenduo Jiang, Yun Hu, Yimin Jia, Ruqian Zhao

**Affiliations:** 1 MOE Joint International Research Laboratory of Animal Health & Food Safety, Nanjing Agricultural University, Nanjing, Jiangsu P. R. China; 2 Key Laboratory of Animal Physiology & Biochemistry, College of Veterinary Medicine, Nanjing Agricultural University, Nanjing, Jiangsu P. R. China

**Keywords:** Chicken, Fatty liver syndrome, FTO, GR, Lipogenesis, m^6^A, YTHDF2

## Abstract

**Background:**

Glucocorticoid receptor (GR) mediated corticosterone-induced fatty liver syndrome (FLS) in the chicken by transactivation of Fat mass and obesity associated gene (*FTO*), leading to demethylation of N6-methyladenosine (m^6^A) and post-transcriptional activation of lipogenic genes. Nutrition is considered the main cause of FLS in the modern poultry industry. Therefore, this study was aimed to investigate whether GR and m^6^A modification are involved in high-energy and low protein (HELP) diet-induced FLS in laying hens, and if true, what specific m^6^A sites of lipogenic genes are modified and how GR mediates m^6^A-dependent lipogenic gene activation in HELP diet-induced FLS in the chicken.

**Results:**

Laying hens fed HELP diet exhibit excess (*P* < 0.05) lipid accumulation and lipogenic genes activation in the liver, which is associated with significantly increased (*P* < 0.05) GR expression that coincided with global m^6^A demethylation. Concurrently, the m^6^A demethylase *FTO* is upregulated (*P* < 0.05), whereas the m^6^A reader *YTHDF2* is downregulated (*P* < 0.05) in the liver of FLS chickens. Further analysis identifies site-specific demethylation (*P* < 0.05) of m^6^A in the mRNA of lipogenic genes, including *FASN*, *SREBP1* and *SCD*. Moreover, GR binding to the promoter of *FTO* gene is highly enriched (*P* < 0.05), while GR binding to the promoter of *YTHDF2* gene is diminished (*P* < 0.05).

**Conclusions:**

These results implicate a possible role of GR-mediated transcriptional regulation of m^6^A metabolic genes on m^6^A-depenent post-transcriptional activation of lipogenic genes and shed new light in the molecular mechanism of FLS etiology in the chicken.

## Background

Fatty liver syndrome (FLS) is a metabolic disease mostly observed in laying hens, which is characterized by increased lipid accumulation in the liver [1]. FLS occurs at the rate between 4% and 20% in chickens kept in intensive systems, which may cause dramatic drop in egg production and increased mortality, leading to considerable economic losses [2, 3]. Several factors have been reported to contribute to the development of FLS, including genetics, environment, nutrition, toxic substances, and hormones [4, 5]. Among these, nutrition is considered the main cause of FLS in the modern poultry industry. Nutritionally over-fed laying hens are at risk for developing FLS [4], and a high-energy maize diet produces a higher incidence of FLS than a low-energy barley diet [6]. Also, high-energy low-protein (HELP) diet is used to establish a model of FLS in some previous publications [7–9].

FLS is caused primarily by an imbalance of hepatic energy influx and efflux. Glucocorticoids (GC) play an important role in hepatic metabolic homeostasis. Chronically elevated GC level is a common feature of fatty liver in humans and animal models [10, 11]. The actions of GC are primarily mediated by glucocorticoid receptor (GR) [12]. Over-activation of GR pathway leads to transcriptional up-regulation of lipogenic genes, causing hepatic steatosis [13]. In chickens, corticosterone (CORT) is the main active form of GC [14]. We have shown previously that excessive CORT administration causes FLS in chickens, which is characterized by excessive lipid accumulation in the liver [15–17]. GR is up-regulated in the liver of FLS chickens [15–17], yet it remains unknown how GR contributes to diet-induced FLS in the chicken.

Three families of protein components are involved in the dynamic m^6^A methylation. The methyltransferases complex formed by methyltransferase-like 3 (METTL3), methyltransferase-like 14 (METTL14), and Wilms’ tumor 1-associating protein (WTAP) [18–20] are “writers” to transfer methyl group to the adenosine of the consensus m^6^A motif in the target RNA. The two demethylases, termed as “erasers”, fat mass and obesity-associated protein (FTO) and α-ketoglutarate-dependent dioxygenase AlkB homolog 5 (ALKBH5) [21, 22], are responsible to remove the methyl group from the m^6^A. The “readers”, such as YTH-domain family 1–3 (YTHDF1–3), can recognize this methylation and regulate the RNA metabolism including stability or translation efficiency of the mRNA [23–26]. m^6^A modification plays a key role in lipid accumulation and energy metabolism [27, 28]. Recently, GR is reported to mediate corticosterone-induced fatty liver in the chicken by transactivation of FTO, leading to demethylation of m^6^A and post-transcriptional activation of lipogenic genes such as sterol regulatory element-binding protein-1 (*SREBP1*), fatty acid synthase (*FASN*) and stearoyl-CoA desaturase (*SCD*) [17]. However, it remains unclear what specific sites of lipogenic genes are demethylated and which m^6^A binding protein is involved in mediating m^6^A-dependent post-transcriptional activation of lipogenic genes.

Therefore, this study was aimed to investigate whether GR and m^6^A modification are involved in HELP diet-induced FLS in laying hens, and if yes, what specific m^6^A sites of lipogenic genes are modified and how GR mediates m^6^A-dependent lipogenic gene activation in HELP diet-induced FLS in the chicken.

## Methods

### Animals and treatment

Forty-eight Hy-Line Variety Brown laying hens (260 days of age, 1.69 ± 0.09 kg in body weight) were raised in the animal house of Nanjing Agricultural university, with the room temperature at approximately 24 °C, and the light regime of 16 L: 8D. Three hens were housed in each cage (60 cm × 46 cm × 44 cm) equipped with a nipple drinker. Hens were randomly divided into control (CON, twenty-four chickens in eight cages) and high-energy low protein diet (HELP, twenty-four chickens in eight cages) groups, fed control diet (2,610 kcal/kg metabolizable energy, 16.9% crude protein) and HELP diet (3,100 kcal/kg metabolizable energy, 12.1% crude protein), respectively, for 12 weeks. The ingredient and calculated composition of the diets used in the current study are presented in Table 1. Hens were subjected to feed restriction (110 g per hen per day) with free access to water throughout the experiment. After 12 weeks of dietary treatment, 1 hen from each cage was randomly selected and killed by rapid decapitation that is considered acceptable for euthanasia of birds according to American Veterinary Medical Association (AVMA) Guidelines for the Euthanasia of Animals: 2013 Edition. Liver samples were rapidly frozen in liquid nitrogen and kept at − 80 °C for further analysis. Among eight hens from HELP group, six hens were diagnosed as FLS according to the hepatic triglyceride (TG) content. So, the sample size was adjusted of both CON and HELP group to six. We conducted a statistical assessment with the replicate number by using G*Power 3.1.9.2 with power (1-β) set at 0.95 and α = 0.05. According to the TG content in the liver (CON = 21.62 ± 2.85 mg/g; HELP = 42.45 ± 7.62 mg/g), we have calculated the effective size d = 3.62. A sample size of 8 participants (4 per group) was needed. Therefore, 12 participants (6 per group) provide sufficient power to study the molecular mechanism underlying the diet-induced FLS in the present study.

**Table 1:** Ingredient and calculated composition of the diets (based on air-dried weight)

Items	CON	HELP
Ingredient, %		
Corn	62.1	68.21
Soybean meal	26	13
Soybean oil	0	6.5
Limestone	8.9	8.9
Lysine	0	0.34
Methionine	0	0.14
Premix^a^	3	3
Total	100	100
Calculated composition, %		
Metabolizable energy, kcal/kg	2,610	3,100
Crude protein	16.9	12.1
Calcium	3.21	3.17
Available P	0.59	0.58
Lysine	0.80	0.77
Methionine	0.38	0.34

^a^The premix was composed of the following per kg diet: VA 9,000 IU; VD_3_, 3,600 IU; VE, 12 IU; VK_3_, 3.00 mg; VB_1_, 2.00 mg; VB_2_, 6.90 mg; VB_6_, 2.70 mg; VB_12_, 0.02 mg; D-Biotin, 0.23 mg; nicotinic acid, 31.20 mg; Folic acid, 1.00 mg; VB_5_, 10.80 mg; Choline chloride, 0.30 g; Fe (as FeSO_4_), 60.00 mg; Cu (as CuSO_4_), 12.00 mg; Mn, 90.00 mg; Zn (as ZnSO_4_), 90.00 mg; I (as KI), 0.80 mg; Se (as Na_2_SeO_3_), 0.30 mg

### Histological evaluation

To visualize the hepatic fat droplets, fresh frozen liver samples were embedded in optimal cutting temperature (OCT) compound and sliced into 8 μm sections. The frozen sections were stained with oil red O (Sigma Aldrich, Saint Louis, MO, USA) for 30 min, counter-stained with H&E for 30 s, then mounted in neutral resin. The slides were observed by using an optical light microscope (Olympus-BX53, Tokyo, Japan).

### Determination of triglyceride content in liver

TG content in liver was measured by using TG assay kits (E1013, Applygen Technologies Inc., Beijing, China) following the manufacturer’s instructions. Briefly, 50 mg of frozen liver sample was homogenized in 1 mL of isopropanol manually in a glass homogenizer with 10 passes on ice, incubated at 4 °C for 10 min. The supernatants were collected and used to measure the hepatic TG contents following the instruction of the TG assay kit.

### Determination of corticosterone content in plasma

CORT content in plasma was measured by using chicken CORT ELISA Kit (E-EL-0160c, Elabscience, TX, USA) following the manufacturer’s instructions.

### Total RNA isolation and real-time PCR

Total RNA was isolated from liver sample (30 mg) using TRIzol Reagent (Invitrogen, Carlsbad, CA, USA) and reverse transcribed into cDNA by using HiScript II Q RT SuperMix for qPCR (+gDNA wiper) (R223–01, Vazyme, Nanjing, China). The coding sequences were used to design specific oligonucleotide primers (GenScript Biotech Co., Nanjing, China) for PCR (Table 2) with AceQ qPCR SYBR Green Master Mix (Q111–02, Vazyme, Nanjing, China) on the Applied Biosystems QuantStudio 6 Flex Real-Time PCR System (Applied Biosystems, Foster City, CA, USA). The relative mRNA abundance was calculated with the 2^−ΔΔCt^ method using GAPDH as an internal reference.

### Protein extraction and Western blotting analysis

Protein was extracted from 40 mg frozen liver sample as previously described [29]. The protein concentration was determined with a Pierce BCA Protein Assay Kit (Thermo Fisher, Waltham, MA, USA). Forty micrograms of protein were used for electrophoresis on the 10% SDS-PAGE gel. Western blot analysis for SREBP1 (AB12162, Abcam, Cambridge, MA, USA, diluted 1:500), SCD (sc-30081, Santa Cruz, CA, USA, diluted 1:200), GR (Custom made for chickens by Genecreate Biotech Co., Wuhan, China, diluted 1:1,000), METTL3 (AB98009, Abcam, diluted 1:1,000), METTL14 (AB98116, Abcam, diluted 1:1,000), FTO (AB77547, Abcam, diluted 1:1,000), YTHDF1 (17479–1-AP, Proteintech, Chicago, IL, USA, diluted 1:2,000), YTHDF2 (24744–1-AP, Proteintech, diluted 1:2,000) and YTHDF3 (25537–1-AP, Proteintech, diluted 1:1,000) were carried out according to the recommended protocols provided by the manufacturers, The density of each protein band was normalized by that of Tubulin α, the internal control. All antibodies were verified to work with chicken samples in previous publications [16, 17]. Images were captured by VersaDoc 4000MP system (Bio-Rad, Hercules, CA, USA) and the band density was analyzed with Quantity One software (Bio-Rad, USA).

### RNA m^6^A dot blot assays

For m^6^A dot blot, 500 ng RNA sample was denatured at 95 °C for 5 min and transferred onto a Hybond-N^+^ membrane (GE Healthcare, Piscataway, NJ, USA). After UV crosslinking, the membrane was washed with TBST buffer, blocked with 5% non-fat milk, and incubated with anti-m^6^A antibody (AB151230, Abcam, diluted 1:1,000) overnight at 4 °C. Then, the membrane was incubated with secondary antibody at room temperature for 2 h. The signals were visualized by the chemiluminescence system (Bio-Rad, USA) and the dot density was analyzed with Quantity One software (Bio-Rad, USA), with staining of 0.02% methylene blue (in 0.3 mol/L sodium acetate, pH = 5.2) as loading control.

### SELECT for detection of m^6^A

From the m^6^A-seq database obtained in a previous study on CORT-induced FLS chickens [17], sequences with m^6^A peaks were retrieved for lipogenic mRNAs including *SREBP1*, *SCD* and *FASN*, and subjected to specific m^6^A site analysis with SRAMP (http://www.cuilab.cn/sramp). One very high/high confidence m^6^A site was selected for each gene and verified by using a single-base elongation- and ligation-based qPCR amplification method (termed as “SELECT”). Briefly, 5 μg total RNA was incubated with 40 nmol/L Up Primer, 40 nmol/L Down Primer and 5 nmol/L dNTP in 17 μL 1× CutSmart buffer (50 mmol/L KAc, 20 mmol/L Tris-HAc, 10 mmol/L MgAc_2_, 100 μg/mL BSA) and annealed under the program as follows: 90 °C (1 min), 80 °C (1 min), 70 °C (1 min), 60 °C (1 min), 50 °C (1 min) and 40 °C (6 min). Next, 17 μL annealing products were incubated with a 3 μL of enzyme mixture containing 0.01 U Bst 2.0 DNA polymerase, 0.5 U SplintR ligase and 10 nmol ATP. The final 20 μL reaction mixture was incubated at 40 °C for 20 min, denatured at 80 °C for 20 min and kept at 4 °C. Quantitative PCR analysis was run under the following conditions: 95 °C, 5 min; (95 °C, 10 s; 60 °C, 45 s) for 40 cycles. The SELECT products of tested site were normalized to the RNA abundance of the mRNA transcript bearing this site. Primers used in SELECT assay are listed in the Table 3.

### Chromatin immunoprecipitation (ChIP) assay

ChIP was carried out as previously described [30]. Briefly, 200 mg frozen liver samples were ground in liquid nitrogen and washed with PBS containing protease inhibitor cocktail (Roche, Basel, Switzerland). After cross-linking in 1% formaldehyde, the reaction was stopped with 2.5 mol/L glycine. The pellets were lysed and chromatin was sonicated to an average length of ∼ 300 bp and the protein-DNA complex was diluted in ChIP dilution buffer, incubated with 2 μg of GR antibody (sc-1004, Santa Cruz, California, USA) overnight at 4 °C. A negative control was included with normal IgG or no antibody. Protein G agarose beads (sc-2003, Santa Cruz, California, USA) were added to capture the immunoprecipitated chromatin complexes. Reverse cross-linking was performed at 65 °C for 5 h to release DNA fragments from the immunoprecipitated complex and DNA was purified. The putative GREs in *FTO*, *YTHDF1* and *YTHDF2* promoters were predicted using JASPAR 2020 (http://jaspar.genereg.net) [31]. Immunoprecipitated DNA was used as a template for real-time PCR. The primers used to amplify the sequences covering these putative GREs are listed in Table 2.

**Table 2:** Nucleotide sequences of primers

Target genes	Primer sequences (5′to 3′)	Used for
*SREBP1*	F: CTACCGCTCATCCATCAACG	Real-time PCR
	R: CTGCTTCAGCTTCTGGTTGC	
*FASN*	F: CGTCATCACCGTCTATC	Real-time PCR
	R: GTAGGCTCCTCCCATC	
*SCD*	F: CTATGCGGGGCTACTT	Real-time PCR
	R: GGATGGCTGGAATGAA	
*GR*	F: CTTCCATCCGCCCTTCA	Real-time PCR
	R: TCGCATCTGTTTCACC	
*METTL3*	F: GCTCCATCCAGGCCCATAAG	Real-time PCR
	R: CCCACTCACCGTATCGATGG	
*METTL14*	F: GTGATTCTCCTGGAGCCACC	Real-time PCR
	R: TGGGGTCCAGAGTCTTCGTT	
*FTO*	F: TGAAGGTAGCGTGGGACATAGA	Real-time PCR
	R: TGAAGGTAGCGTGGGACATAGA	
*YTHDF1*	F: ACAAGCGTTGACCCTCAGAGA	Real-time PCR
	R: TGTTCCCCAAGCTGAGAAGG	
*YTHDF2*	F: TCCTACTCTCTGGGTGAGGC	Real-time PCR
	R: GCGTAATTGCTGCTGTAGCC	
*YTHDF3*	F: CCACCAACACTGGTGCAAAG	Real-time PCR
	R: GCCCACACCCCTATTACGAG	
*FTO Fragment*	F: AAAACTGAGGGGGGAT	ChIP PCR
	R: ACAACTGTGGGCAAGG	
*YTHDF2 Fragment 1*	F: GTGCTTGTTGCTACTCGT	ChIP PCR
	R: CCATAGAGGAACCCAATC	
*YTHDF2 Fragment 2*	F: GGGCTCAGGTGGTTTGTT	ChIP PCR
	R: CACGTCCAGCGATTCATC	
*YTHDF1 Fragment*	F: GCAGGTATTTTGACACTT	ChIP PCR
	R: TATGCATTGACCAGAACT

**Table 3:** Nucleotide sequences of SELECT method

Target	Sequences (5’to 3′)
*SREBF1 3’UTR X site*	Up Probe: tagccagtaccgtagtgcgtgCCCATTGGTTTCGGAAAGAG
	Down Probe: CCCCTTTGGTGGCACGACGGcagaggctgagtcgctgcat
*SREBF1 3’UTR N site*	Up Probe: tagccagtaccgtagtgcgtgCACGACGGGGTCCCGCTGGA
	Down Probe: CGGCGAGAGGGTCCCACTCAcagaggctgagtcgctgcat
*SCD 3’UTR X site*	Up Probe: tagccagtaccgtagtgcgtgCTTGTGACTCCCATCTCCAG
	Down Probe: CCGCATTTTCCGGGCCAAGAcagaggctgagtcgctgcat
*SCD 3’UTR N site*	Up Probe: tagccagtaccgtagtgcgtgTTTTCCGGGCCAAGATGACC
	Down Probe: CCTTGGAGACCTTCTTGCGAcagaggctgagtcgctgcat
*FASN 3’UTR X site*	Up Probe: tagccagtaccgtagtgcgtgGTGCTCCAGGATTATCTCAG
	Down Probe: TCTTCTTTCTTAATGTTATTcagaggctgagtcgctgcat
*FASN 3’UTR N site*	Up Probe: tagccagtaccgtagtgcgtgATGGCGATGAGAAGCCGTGC
	Down Probe: CCAGGATTATCTCAGTTCTTcagaggctgagtcgctgcat
qPCR	Forward Prime: ATGCAGCGACTCAGCCTCTG
	Reverse Prime: TAGCCAGTACCGTAGTGCGTG

### Statistical analysis

Differences between two groups were analyzed by t-test using SPSS 20.0 software (SPSS Inc., Chicago, IL, USA). Data are expressed as means ± SEM. Pearson correlation analysis was performed for correlation analysis. The differences were considered statistically significant when *P* < 0.05.

## Results

### Lipogenic genes are activated in the liver of chickens fed HELP diet

**Fig. 1:**
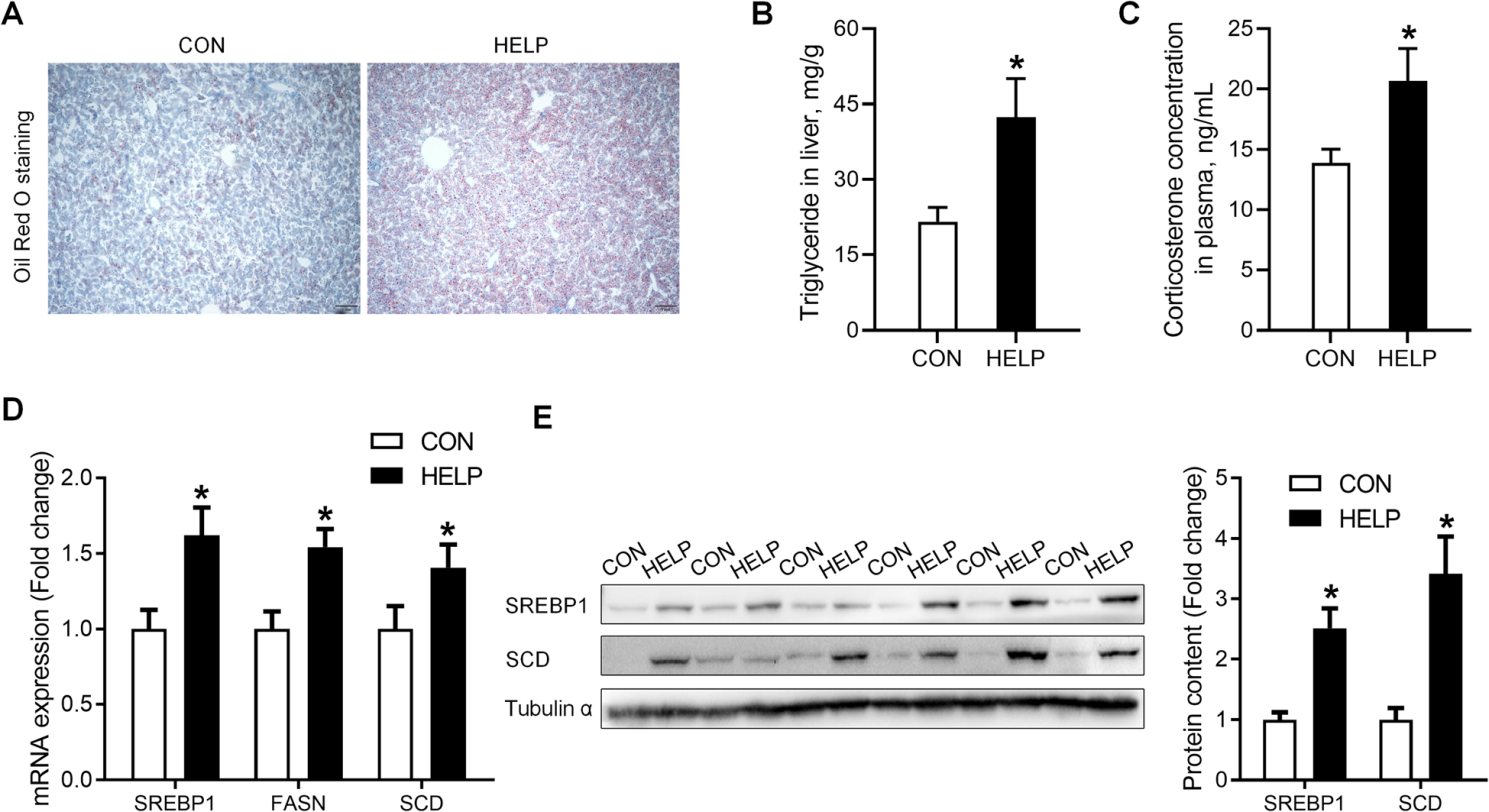
Lipogenic genes are activated in the liver of chickens fed HELP diet. **A** histological sections stained with oil-red, *n* = 3; **B** liver concentration of TG, *n* = 6; **C** the plasma corticosterone level, *n* = 6; **D** mRNA expression of *SREBP1*, *FASN* and *SCD* with qPCR, *n* = 6; **E** protein content of SREBP1 and SCD with western blot, *n* = 6. CON = Control; HELP = High-energy low-protein. Values are means ± SEM, *P < 0.05

Chickens fed HELP diet had significantly higher hepatic lipid accumulation compared with their control counterparts, as seen in Oil Red O staining (Fig. 1A) and hepatic TG (Fig. 1B) content (*P* < 0.05). Meanwhile, plasma CORT concentration was significantly elevated (*P* < 0.05) in HELP group (Fig. 1C). Moreover, hepatic expression of lipogenesis genes, such as *SREBP1*, *FASN* and *SCD* were significantly up-regulated (*P* < 0.05) at both mRNA (Fig. 1D) and protein (Fig. 1E) levels.

### HELP diet increases hepatic GR expression and decreases global RNA m^6^A methylation

**Fig. 2:**
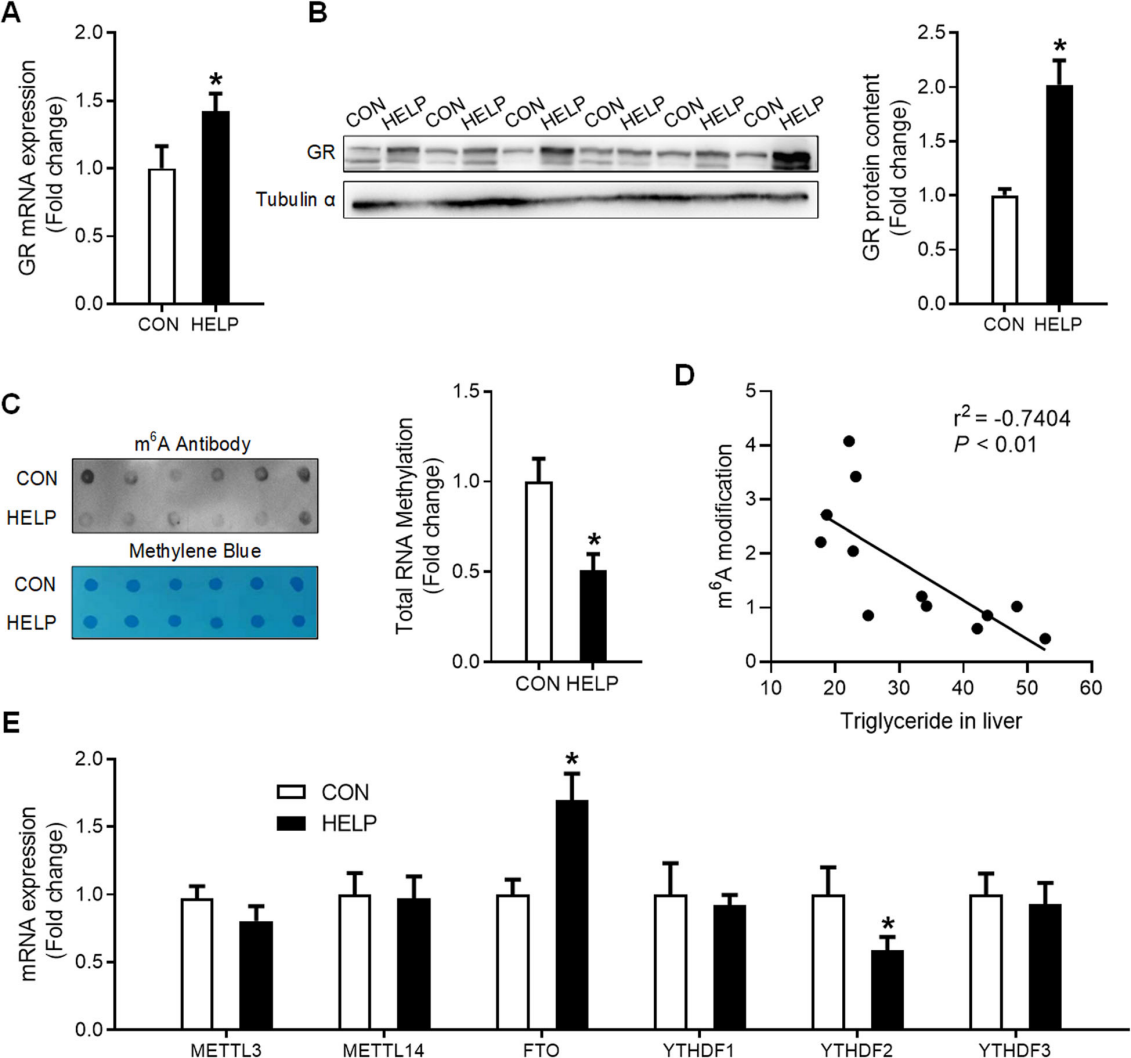
HELP diet increases hepatic GR expression and decreases global RNA m^6^A methylation. **A** and **B** protein content and mRNA expression of GR with western blot and qPCR, *n* = 6; **C** global RNA m^6^A level in the liver was detected by dot-blot, *n* = 6; **D** correlation analysis was performed between TG concertation and m^6^A modification; **E** mRNA expression of the RNA methyltransferases METTL3 and METTL14, the m^6^A demethylase FTO, and the reader proteins YTHDF1 and YTHDF3 were detected with qPCR, *n* = 6. CON = Control; HELP = High-energy low-protein. Values are means ± SEM, *P < 0.05

**Fig. 3:**
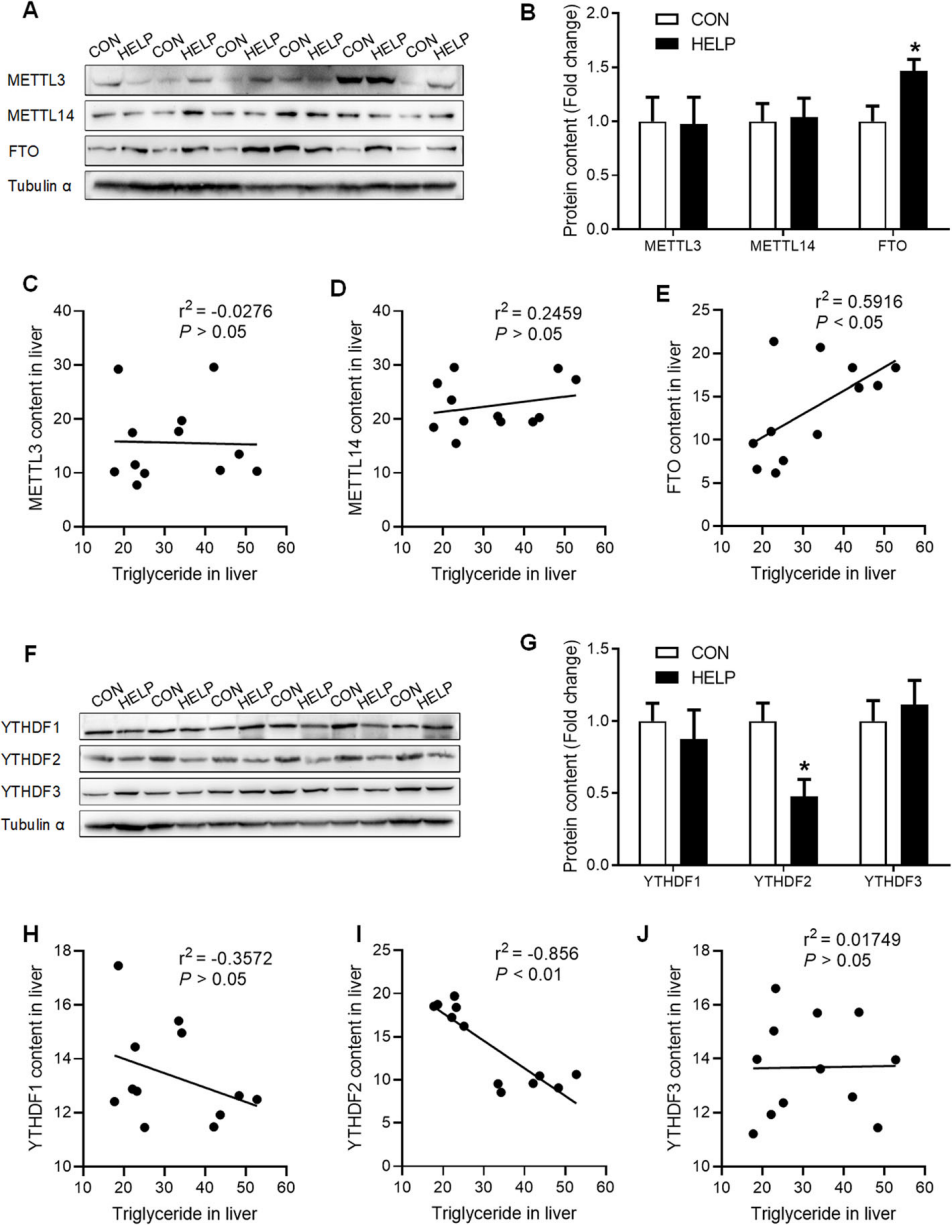
HELP diet increases hepatic FTO and YTHDF2 protein content. **A** and **B** protein content of METTL3, METTL14, and FTO in the liver were detected by western blot, *n* = 6; **C**-**E** correlation analysis was performed between TG concertation and METTL3, METTL14, FTO, *n* = 6; **F** and **G** protein content of YTHDF1, YTHDF2 and YTHDF3 in the liver were detected by western blot, *n* = 6; **H**-**J** correlation analysis was performed between TG concertation and YTHDF1–3, *n* = 6. CON = Control; HELP = High-energy low-protein. Values are means ± SEM, *P < 0.05

GR was significantly increased (*P* < 0.05), at both mRNA (Fig. 2A) and protein (Fig. 2B) levels, in the liver of chickens fed HELP diet. Meanwhile, HELP diet significantly decreased (*P* < 0.05) mRNA m^6^A levels (Fig. 2C) in the liver. Hepatic TG contents were negatively correlated to global m^6^A levels (r^2^ = − 0.7404, *P* < 0.01, Fig. 2D). No significant alterations were detected for the expression of RNA methyltransferases (METTL3 and METTL14) or the reader proteins (YTHDF1 and YTHDF3) (Figs. 2E, 3A-B, F-G), and no correlation found between hepatic TG contents and METTL3, METTL14, YTHDF1 and YTHDF3 protein content in liver (Fig. 3C-D, H, J). However, RNA demethylase FTO (Figs. 2E, 3A-B) was significantly increased (*P* < 0.05), and the reader protein YTHDF2 (Figs. 2E, 3F-G) was significantly decreased (*P* < 0.05) in the liver of chickens fed HELP diet. Moreover, hepatic TG contents are positively correlated to FTO protein content (r^2^ = 0.5916, *P* < 0.05, Fig. 3E) and negatively correlated to hepatic YTHDF2 protein content (r^2^ = − 0.856, *P* < 0.01, Fig. 3I) in FLS hens.

### Levels of m^6^A on specific sites of lipogenic mRNAs are decreased in the liver of HELP diet-fed chickens

**Fig. 4:**
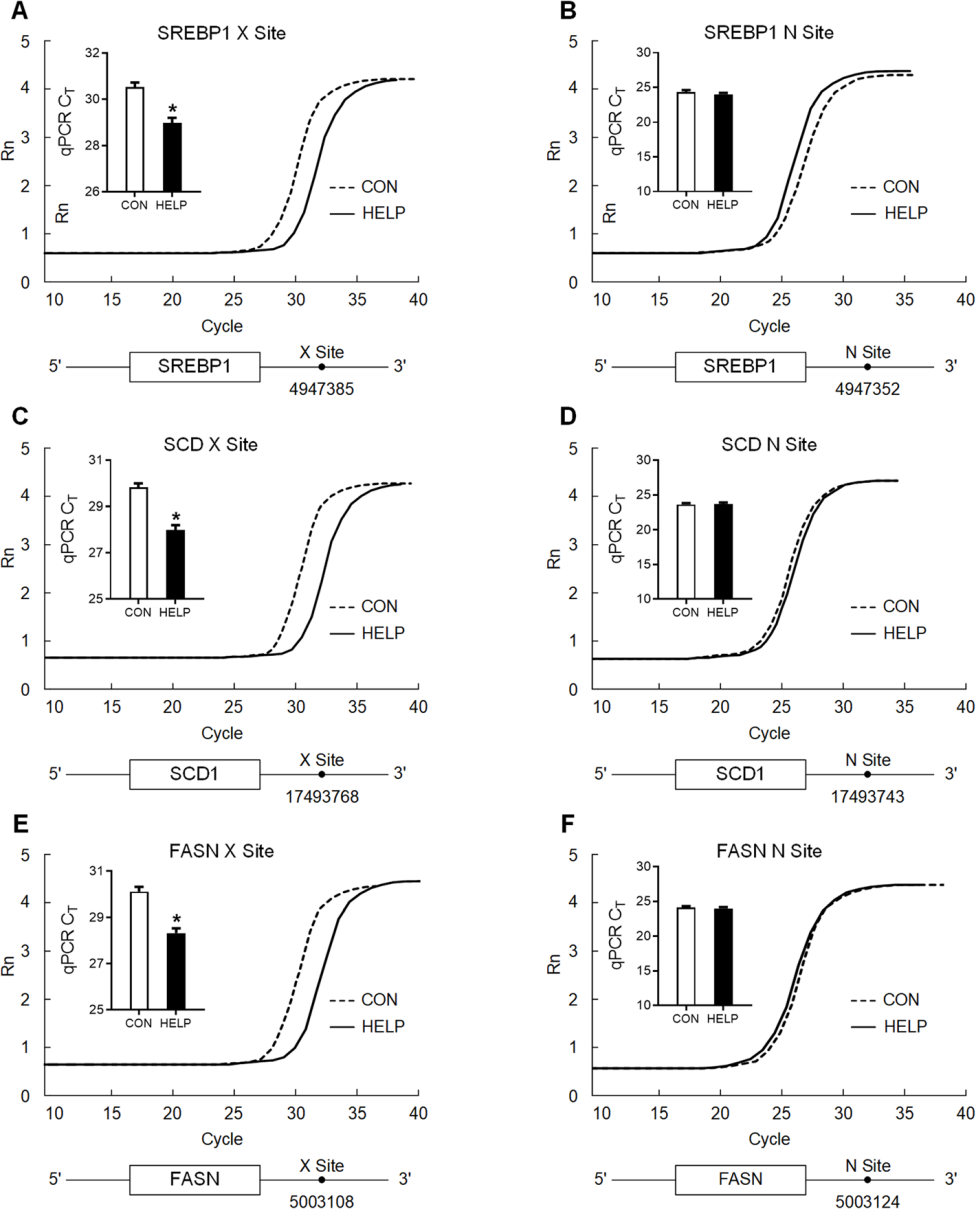
3’UTR of lipogenic mRNAs is m^6^A hypermethylated in the liver of HELP diet-fed chickens. **A** and **B** detection of m^6^A modification in *SREBP1* 3’UTR using SELECT, *n* = 6. X site was predicted by SRAMP and N site (non-modification site) was negative control. **C** and **D** detection of m^6^A modification in *SCD* 3’UTR using SELECT, *n* = 6. X site was predicted by SRAMP and N site (non-modification site) was negative control. **E** and **F** detection of m^6^A modification in *FASN* 3’UTR using SELECT, *n* = 6. X site was predicted by SRAMP and N site (non-modification site) was negative control. CON = Control; HELP = High-energy low-protein. Values are means ± SEM, *P < 0.05

Specific m^6^A sites on 3’UTR of *SREBP1*, *SCD* and *FASN* mRNAs were selected for site-specific m^6^A quantification by SELECT method. Cycle threshold numbers were significantly increased (*P* < 0.05) at potential m^6^A site (X site, Fig. 4A, C and E), but not at the negative control site (N site, Fig. 3B, D and F), indicating decreased m^6^A modification on specific site of lipogenic mRNA 3’UTRs in the liver of HELP diet-fed chickens.

### GR binding to the promoter of *FTO* and *YTHDF2* genes is modulated in the liver of HELP diet*-*fed chickens

**Fig. 5:**
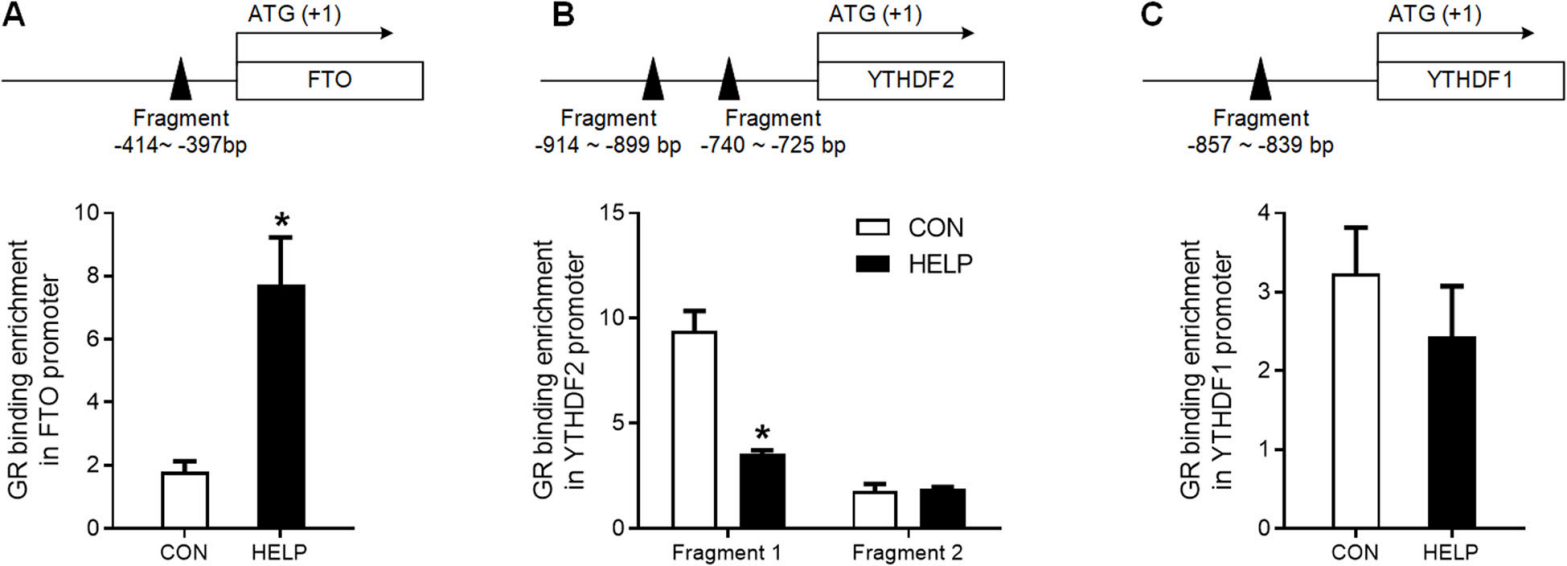
GR binding to the promoter of *FTO* and *YTHDF2* genes is modulated in the liver of HELP diet*-*fed chickens. **A** schematic representation of GR transcription factor binding promoter of *FTO* (up) and ChIP-PCR assay was used to measure the binding of GR on *FTO* promoter in the liver of HELP diet-fed chickens (down, *n* = 3). **B** schematic representation of GR transcription factor binding promoter of *YTHDF2* (up) and ChIP-PCR assay was used to measure the binding of GR on *YTHDF2* promoter in the liver (down, *n* = 3). **C** schematic representation of GR transcription factor binding promoter of *YTHDF1* (up) and ChIP-PCR assay was used to measure the binding of GR on *YTHDF1* promoter in the liver (down, *n* = 3). CON = Control; HELP = High-energy low-protein. Values are means ± SEM, *P < 0.05

ChIP-PCR analysis revealed changes of GR binding on the promoter of *FTO* and *YTHDF2* genes. Fragments containing putative GREs on the promoter of *FTO* (Fig. 5A), *YTHDF2* (Fig. 5B) and *YTHDF1* (Fig. 5C) were amplified after chromatin immunoprecipitation with GR antibody. GR binding to the fragment of *FTO* gene promoter (Fig. 5A) was significantly increased (*P* < 0.05), while that to the fragment 1 of *YTHDF2* gene promoter (Fig. 5B) was significantly decreased (*P* < 0.05), in the liver of HELP diet-fed chickens. In contrast, GR binding to the fragment 2 of *YTHDF2* or *YTHDF1* gene promoter was not affected.

## Discussion

Accumulating evidences indicate that FTO-dependent RNA demethylation and nonalcoholic fatty liver disease are closely intertwined [32, 33]. FTO-dependent demethylation of m^6^A leads to an increase in lipogenic expression in hepatocytes through m^6^A modification [34, 35]. Previously, we used chronic administration of corticosterone (CORT) to establish an *in vivo* FLS model in the juvenile chickens, and to induce excessive lipid accumulation in primary chicken hepatocytes *in vitro* with combined treatment of oleic acid and dexamethasone (OA/DEX) [17]. In both *in vivo* and *in vitro* models, we found that GR-mediated transactivation of FTO and m^6^A demethylation contribute to lipogenic gene activation. Interestingly, HELP diet-induced FLS is also associated with global m^6^A demethylation and the activation of lipogenic genes in the liver of laying hens. It may not be totally unexpected, because all these fatty liver models, no matter how they are induced, share the same hormonal and biochemical status of elevated CORT and lipid concentration in the blood.

The major mechanism by which m^6^A exerts its effects is recruiting m^6^A-binding proteins [36]. m^6^A can be recognized by proteins that contain a YTH (YT521B homology) domain [37] or alternatively by eukaryotic initiation factor 3 (eIF3) [38, 39]. The functions of m^6^A-binding proteins are context-dependent, which means that different m^6^A-binding proteins bind m^6^A on different regions of mRNA to exert different functions in gene regulation [40]. Among three m^6^A-binding proteins determined in the present study, YTHDF2 was down-regulated at both mRNA and protein levels, indicating YTHDF2-mediated gene regulation. *YTHDF2* binds transcripts carrying m^6^A in 3’UTR to induce mRNA degradation partially through recruiting the CCR4-NOT deadenylase complex [41]. *YTHDF2* was reported to selectively recognize m^6^A sites in *FASN* mRNA, leading to increased *FASN* mRNA decay and decreased FASN protein content in HepG2 cell lines [42]. Previously, we conducted a m^6^A-seq analysis to elaborate the epitranscriptomic modification of m^6^A in the liver of CORT-induced FLS chickens [17]. From this published database, we selected some CORT-responsive m^6^A peaks in 3’UTR of the lipogenic transcripts, and identified specific HELP-responsive m^6^A sites on 3’UTR of lipogenic mRNAs, including *SREBP1*, *SCD* and *FASN*, with SELECT analysis. It is possible that the down-regulation of *YTHDF2* impairs m^6^A-dependent lipogenic mRNA degradation, which leads to augmented lipogenesis and excessive lipid accumulation in the liver of FLS hens.

The molecular mechanism by which HELP-diet induces hepatic up-regulation of *YTHDF2* in the chicken is unknown. *YTHDF2* is negatively regulated by miR-145/miR-495/miR-493-3p at post-transcriptional level in hepatocellular carcinoma cells and prostate cancer cells [43–45]. Moreover, *YTHDF2* can be SUMOylated *in vivo* and *in vitro* at the site of K571, which significantly increases its binding affinity with m^6^A-modified mRNAs [46]. HIF-2α is reported to transrepress YTHDF2 in hepatocellular carcinoma cells [47]. Based on the observation that YTHDF2 is down-regulated at both mRNA and protein level, we come up with a hypothesis that GR may directly transactivate YTHDF2. Silicon analysis using JASPAR online database identifies 2 putative GR binding sites for YTHDF2. These binding sites were then functionally validated using ChIP-PCR. Indeed, GR binding to *YTHDF2* gene promoter is decreased in the liver of hens fed HELP diet. It remains a mystery how GR binding to the promoter of *FTO* and *YTHDF2* genes is distinctively regulated, leading to FTO up-regulation and YTHDF2 down-regulation in HELP diet-induced FLS. Some unidentified co-factors must come into play to coordinate the down-stream effectors of GR action.

In this study, GR was upregulated at both mRNA and protein levels in the liver of hens fed HELP diet. The altered GR binding to the promoter of *FTO* and *YTHDF2* genes indicate HELP diet-induced modulation in GR activation. GR can be activated via both ligand-dependent [48] and ligand-independent manners [49]. Previously, we reported CORT-dependent GR activation in the liver of CORT-treated chickens [16, 50]. In this study, chickens are not treated with CORT and the plasma CORT level was not determined. Therefore, we cannot draw a conclusion whether the altered GR expression and binding is dependent on CORT. Nevertheless, GR can be activated by cellular stressors through p38 MAPK-mediated phosphorylation of Ser134, which is a hormone-independent phosphorylation site on the human GR [51]. GR can also be activated by various stimuli in the absence of glucocorticoid ligands, such as elevated temperature, excessive inflammation, and cancer [52–54]. Therefore, both ligand-dependent and ligand-independent pathways are possible in HELP diet-induced alteration in hepatic GR activation.

## Conclusions

The present results have shown that GR-mediated transcriptional regulation of *FTO* and *YTHDF2* contributes to lipogenic gene activation by site-specific demethylation in HELP diet-induced chicken FLS. These findings add YTHDF2-mediated m^6^A modification as a new component of GR signaling in the regulation of fat metabolism in the liver and shed new light on developing effective therapeutic strategies in the prevention and treatment of HELP diet-induced chicken FLS.

## Data Availability

The datasets used and analyzed during the current study available from the corresponding author upon request.

## Abbreviations

ALKBH5: α-ketoglutarate-dependent dioxygenase AlkB homolog 5
ChIP: Chromatin immunoprecipitation
CORT: Corticosterone
DEX: Dexamethasone
eIF3: Eukaryotic initiation factor 3
FASN: Fatty acid synthase
FLS: Fatty liver syndrome
FTO: Fat mass and obesity associated gene
GC: Glucocorticoids
GR: Glucocorticoid receptor
HELP: High-energy low-protein
m^6^A: N6-methyladenosine
METTL3: Methyltransferase-like 3
METTL14: Methyltransferase-like 14
OA: Oleic acid
SCD: Stearoyl-CoA desaturase
SELECT: Single-base elongation- and ligation-based qPCR amplification method
SREBP1: Sterol regulatory element-binding protein-1
TG: Triglyceride
WTAP: Wilms’ tumor 1-associating protein
YTHDF1–3: YTH-domain family 1–3
